# “I hope I die. That is what I hope for”: Qualitative study of lived experiences of mental health of Indian women living with HIV experiencing intersectional stigma

**DOI:** 10.1371/journal.pgph.0002075

**Published:** 2023-12-27

**Authors:** Reshmi Mukerji, Jenevieve Mannell, David Osrin

**Affiliations:** Institute for Global Health, University College London, London, United Kingdom; Thompson Rivers University, CANADA

## Abstract

Poor mental health due to stigma and discrimination has been well documented among women living with HIV. Although they often have other marginalized and stigmatized identities, little is known about their mental health as a result of experiencing multiple stigmas. Current narratives of mental health as a result of HIV-related stigma center on common mental health disorders such as anxiety and depression. However, biomedical diagnostic categories may not be as well known in all cultural and social contexts, and people may choose to express their distress in their own language. It is therefore important to listen to how women express their mental health concerns in their own language—their lived experiences—in order to best support them. To fill this research gap, semi-structured interviews were conducted in Kolkata, India, with 31 women living with HIV and 16 key informants. Data were coded and analyzed using thematic network analysis. The results showed that women suffered from poor mental health, which in turn affected their physical health. This happened through reduced adherence to medication, lowered CD4 counts, and the physical effects of stress, which could be perceived as prolonged. Participants described women’s mental health concerns as worry, sadness, hopelessness, and fear, but biomedical diagnostic labels were rarely used. This allowed women to avoid additional stigmatization due to mental illness, which can attract some risk in this social context. As many women living with HIV experience poor mental health, they should be supported with a combination of psychosocial and psychological interventions. These include screening all women for mental illness and offering them mental health first aid. Those requiring additional support should be offered specialist psychotherapeutic and pharmacological care. This must be accompanied by stigma reduction interventions if they are to be successful in addressing the mental health needs of women living with HIV.

## Introduction

India is currently home to over two million people living with HIV, approximately half of whom are women [1]. This is important because HIV is heavily stigmatized and the experience of stigma is gendered [2]. In his seminal work, Goffman conceptualized stigma as an “attribute that is deeply discrediting” and reduces the bearer “from a whole and usual person to a discounted, tainted one” [3]. Along with their gender, women living with HIV often have other marginalized identities, such as ethnicity and sexual orientation, that are themselves stigmatized [4, 5]. A marginalized identity is an identity that results or has historically resulted in discrimination or negative treatment of a person because of that identity. As people with such identities are often stigmatized and subjected to interpersonal and structural discrimination, people with multiple marginalized identities often experience intersectional stigma, which has been defined as a “mutually constitutive relationship between social identities and structural inequalities” [5]. In the Indian context, identities such as widowhood, having girl children, being a sex worker, and poverty contribute to the stigma experienced by women living with HIV [6]. HIV-related stigma is known to negatively affect women’s mental health in India [7] and elsewhere [8], but little is known about how women living with HIV understand their mental health and describe their feelings as a response to intersectional stigma.

This is a complex issue that might be simplified by a framework for intersectional stigma. One such framework, developed by Turan et al., shows how the stigma of identities such as race, gender, sexuality, and class interacts with HIV-related stigma to affect the mental health of people living with HIV [9]. The model proposes that people with stigmatized identities experience greater stress which ultimately affects HIV outcomes. This happens through different pathways (interpersonal factors, mental health, biological stress processes, and compromised psychological resources) that lead to poor engagement in care and ultimately worsen HIV-related heath [9]. We use this framework to guide our understanding of how experiences of intersectional stigma shape the mental health of women living with HIV, and whether they have an impact on their physical health.

Current narratives of mental health as a result of experiences of HIV stigma and discrimination center on common mental health disorders, such as anxiety and depression [10]. The HIV-related mental health literature from India focuses on such diagnostic categories [11–16]. However, stress and depression present differently in different cultural and social contexts and do not necessarily fit with established diagnostic categories [17, 18]. People may present with body ache or general weakness which are interpreted by healthcare providers as “vague” and not related to mental distress, when in reality their symptoms are expressions of distress [17]. Such “cultural idioms of distress” are considered culturally appropriate ways of communicating distress in circumstances that do not allow for other forms of expression. More importantly, they allow people to articulate their distress, based on their own understanding of mental health, in non-stigmatizing language [19, 20]. It is, therefore, important to listen to how people choose to express their distress in their own language—their lived experiences—in order to understand and best support them. Only a few studies from India have reported on the lived experiences of mental health of women as a result of HIV stigma. These studies have highlighted a range of feelings, including anger, hopelessness, thoughts of death, and guilt and fear [21–23]. Although such expressions may map onto biomedical concepts of common mental health disorders, such as depression or anxiety, the descriptions of mental health experiences in these studies help us understand what women actually feel and the support that they need after experiencing HIV-related stigma and discrimination, without giving them the additional label of “mentally ill”. Mental illness is severely stigmatized in India and has severe consequences, especially for women [24], and we need to acknowledge the importance of understanding women’s mental health in the language that they choose to express themselves. An understanding of such lived experience, which allow women to describe their experiences and feelings based on their own understanding of it is important because (i) it helps us understand women’s mental health experiences in their own (non-stigmatizing) language, which (ii) allows us to acknowledge alternative modes of expression of distress, and (iii) see how women’s experiences might differ from or align with interpretations by healthcare providers. This can be used to inform future clinical practice by encouraging the use of non-stigmatizing mental health language, which otherwise might be especially problematic for multiply stigmatized groups, such as women living with HIV.

If there is little evidence from India and elsewhere of the lived experiences of mental health as a result of HIV stigma, even less is known about the mental health effect of HIV stigma combined with the stigma of other marginalized social identities. We try to fill this gap by providing an account of how women living with HIV in India experience mental health as a result of their experiences of intersectional stigma and related violence, and how that in turn affects their physical health.

## Methods

### Setting

This work was part of a study conducted in 2020–2021 on the intersectional stigma of HIV, domestic violence, and marginalized identities. The methods have been described in detail elsewhere (6). The study was conducted in Kolkata, capital of India’s eastern state of West Bengal. West Bengal was a suitable setting because of its high HIV incidence rate [25] and India’s highest number of reported cases of domestic violence [26]. Interviews with women living with HIV were conducted at an NGO that offered treatment and support services to people living with HIV, and catered to people residing both in and outside Kolkata. Women who accessed services were mostly of lower socioeconomic status, from diverse religious backgrounds, and had either rural or urban residence.

### Design

Semi-structured qualitative interviews were conducted with 31 women living with HIV to understand their experiences of mental health. In addition, 16 key informant interviews were conducted with service providers in an attempt to draw out their interpretations of women’s lived experiences of mental health.

### Data collection

Semi-structured interviews were conducted with a purposive sample of 31 women living with HIV. The sample included only women living with HIV, so that the stigma of HIV and gender was common to all participants. In addition, the sample was chosen to include a range of stigmatized identities such as widowhood, sex work, or having daughters, so that the intersection of multiple stigmas could be captured. Interviews were also conducted with 16 key informants recruited using a combination of personal networks and snowballing. Recruitment continued until saturation such that no new information was emerging from additional interviews. None of the participants dropped out after recruitment. Two key informants refused to participate, after initially agreeing, citing issues of time and signing consent forms.

Interviews with women living with HIV were conducted face-to-face in a private room on the premises of the NGO, with only the interviewer and the participant present to maintain confidentiality. Women were invited when they were attending an outpatient clinic, visiting the NGO for other activities, or because they lived on the premises. A counselor or outreach worker made first contact with clients. If they agreed to be interviewed, they were invited to speak to the researcher, who explained the project in greater detail and asked them to sign a consent form. Considering the sensitivity of the topic, rapport was established through regular visits to the NGO and talking to participants before the interview to make sure they were comfortable with the process. Key informants were interviewed online. They were invited by phone or email and sent a consent form for signed return before the online interview. An interview topic guide explored domestic violence, intersectional stigma, mental health, and coping. Interviews were conducted by author RM: a woman with a Masters in Public Health who was pursuing a PhD degree at the time. Her interest in the topic stemmed from her interest in health-related stigma. She was trained in sensitive interviewing and had previous experience conducting qualitative interviews with women. Participants were informed about the researcher’s background, personal goals, and the goals of the study. The interviews were conducted in Bengali and lasted from 30 minutes to over two hours. Interviews were audio-recorded and detailed field notes prepared after each. After the first two interviews, the researcher went through the recordings and notes and amended the topic guide to include new probes and highlight topics of interest. Field notes were used during analysis to add to the richness of the data by referring to the context of each interview, such as the interview environment to make note of non-verbal cues and emotions expressed by participants, and anything said that was not recorded. Audio recordings were transcribed and translated into English. We generally translated interviewees’ statements verbatim. In accounts of stigma and mental health, we retained the original language (or English equivalents when there was clear correspondence) in order to avoid reifying abstract concepts. Transcripts were de-identified and stored in an encrypted laptop and secure server.

### Data analysis

Data were coded by RM in Nvivo 12.6.1 qualitative software (QSR International) and involved a combination of deductive and inductive coding. The methodological orientation was interpretive phenomenology as a study about the lived experiences of mental health of individuals who have experienced intersectional stigma within a particular social and cultural context (6). Turan et al.’s conceptual framework on intersectional stigma and mental health was used as the theoretical basis for the deductive coding scheme (9). Thematic Network Analysis was used to develop themes from the data [27]. The steps of analysis involved (i) coding of textual data to develop basic themes, (ii) development of middle-order “organizing themes” with shared meaning, and (iii) a final overarching level of analysis to develop a “global theme”. Reflexivity was maintained by the researcher throughout the data collection and analysis process by being aware of her own positionality vis-à-vis the participants, such as race, gender and age, and how it might influence the data and interpretation of results [28], and being aware of her personal biases and beliefs and their possible impact on the results [29]. This was done by referring to field notes, reflecting on the interviews, and maintaining detailed annotations and memos throughout the coding process.

### Ethical considerations

The study received approval from the University of London (UCL) Research Ethics Committee (18291.001) and the head of the partner NGO (approval was granted after having discussions about the study and reviewing all topic guides and consent forms, so that they were aware of the study design and any ethical implications). All participants were taken through an informed consent process and written informed consent was obtained. Participants were informed that their participation was voluntary, all of their information would be treated as confidential, were explained the risks of participation (recounting painful memories), and that not agreeing to take part would not affect the care they received at the NGO. We followed the protocol developed by the World Health Organization for research on violence against women [30]. Interviews were conducted in a non-judgmental manner, participants were offered breaks, and the option to discontinue if there were any signs of distress. The interviewer made sure to end each interview on a positive note and to spend time talking to participants after the conclusion of the actual interview. The counseling staff at the NGO and the interviewer followed up with participants for up to a week after the interviews for signs of potential distress. Findings were shared with participants and partner NGO.

### Inclusivity in global research

Additional information regarding the ethical, cultural, and scientific considerations specific to inclusivity in global research is included in the S1 Checklist.

## Findings

Women had a median age of 35 years and had been living with HIV for an average of 8 years. The majority were seroconcordant and were primarily Hindu, one third were widows, and they were a mix of rural and urban residents of West Bengal. While all women had experienced the stigma of HIV and gender, the additional stigmas that women could be subjected to are captured in Table 1. These identities contributed to their experiences of HIV-related stigma and affected their mental health. Two central themes identified from the data were that *intersectional stigma resulted in poor mental health* of women living with HIV with other marginalized identities, and women felt that the *stress of intersectional stigma led to poor physical health*.

**Table 1:** Additional marginalized identities for women living with HIV.

Marginalized identities	N = 31 (%)
Sex worker	5 (16)
Widowhood	9 (29)
Separated/Single	9 (29)
Remarried	2 (6)
Only daughter(s)	8 (26)
Disability	1 (3)
Religious minority	6 (19)

A single woman could report multiple marginalized identities.

## Intersectional stigma results in poor mental health

Adverse experiences of intersectional stigma of HIV and other marginalized social identities resulted in poor mental health of women living with HIV. In their own words, they reported feeling emotionally distressed by the experiences of stigma and violence, profound sadness, hopeless, and constant worry. Since non-disclosure or strategic disclosure was a common strategy women used to avoid stigmatizing experiences, fear of anticipated stigma if people found out was a real concern and caused considerable stress.

### ‘I am just not able to take this’

Many key informants described women’s experiences of intersectional stigma and related violence in terms of a clinical diagnosis of depression and post-traumatic stress. A key informant from an HIV NGO made the following observation when asked about women’s mental health:

“Depression and trauma, one thing is that she is helpless, so that makes her vulnerable in society as well as in her family… she is not… getting that mental support within the family… anything can be discussed in front of her, which is very abusive for her, so I think it [causes] mental trauma”

(CL002, Official HIV NGO).

Women used different language to express similar narratives of distress due to intersectional stigma. They used terms such as *chinta* (worried or troubled) or *mon bhenge giyeche* (mentally or emotionally broken) and hurt due to experiences of intersectional stigma. They often described how they suppressed their pain after experiencing stigmatizing behaviors. An elderly widow who felt helpless described how she just quietly accepted the stigma-related abuse: “Whatever pain they give me I stay quietly and tolerate, what else to do [crying]” (CL022, Age 51 years). A serodiscordant woman described her experience of intersectional stigma of HIV, gender and domestic violence—she was not believed about the source of infection, her violent husband’s actions were justified, and her status made public by in-laws. Such stigmatizing behaviors made her stress unbearable:

“They tell me I heard that you have this AIDS disease, it hurts to hear all that. I had the disease, but I was dealing with it on my own, I was working, I was fine, there were no worries. Now I cannot sleep at night, I don’t have an appetite, a mental tension has entered into me… You know then my heart just broke. That I would live, what would I fight to live for? I used to be quite plump before, I used to work, but I didn’t have to worry, but now as the days go by it’s like so much worry has entered my mind. What will happen? It’s like my mind is not working. The way they treat me. They even beat me”

(CL002, 30 years).

### ‘I feel very, very sad’

Key informants expressed women’s suffering in terms of mental health problems. An HIV nurse said that women without family support after diagnosis “have a huge amount of mental health problems.” Nevertheless, women did not see this sadness and suffering as mental health problems, but expressed their profound sadness using language such as *dukkho* (sadness) or *koshto* (pain) and hopelessness amidst the stigma and violence they experienced.

Sadness was worse among those with other marginalized identities such as widows, sex workers, or women in greater poverty. Some widows were affected by thoughts that intersectional stigma meant that no one would care for them when they became sick. Others described moments of hopelessness and feelings of extreme loneliness as the loss of their partners combined with the isolation and abuse that resulted from HIV stigma:

“Yes, won’t I feel sad? [smiles] Of course I feel sad. I feel very sad. Alone, completely alone. Even when I go out on the streets I feel alone. That I am alone”

(CL022, Age 51 years).

Younger widows were also affected by the social restrictions placed upon them by widowhood. They were taunted by their in-laws to get married again, knowing this was unlikely given the stigma of HIV and of widow remarriage in India. One woman reminisced about old times and described her sadness at not being able to dress up for social occasions:

“But because I don’t have my husband, so I can’t dress up like married women do… So when I see others like that it makes me sad, I used to wear *Benarasee* sarees, I used to dress up, used to wear different colored blouses [laughs] and heavy jewellery, some heavy makeup, I think about that. I think that even if husband is a drunk or whatever [you can do these if he is alive]”

(CL013, Age 33 years).

Sex workers described their feelings of hopelessness at not only aging, but having to leave their profession because of HIV. When asked about her hopes for the future, one said, “What hope will I see? I don’t have any hope. All my hopes are gone” (CL026, 45 years). Another just answered, “I don’t find mental strength, *didi*” (CL024, 50 years).

### ‘If only God took me away right now’

Some women discussed wanting to die in the initial period after HIV diagnosis. The reasons for this ranged from fears of contracting a life-threatening illness, often termed a “big disease”, to fears of anticipated stigma, to the inability to cope with actual experiences of intersectional stigma and related violence from partners and in-laws. The burden of their adverse experiences of multiple stigmas and related violence was such that they did not want to go on living. Some healthcare providers had a shared understanding with women about how they felt after experiences of intersectional stigma, which they attributed to the stigma-related violence that the women were experiencing rather than just mental health issues:

“This ART that the patient has to take, I mean regularity, the medicine that she must take to stay alive, maybe she is forgetting to take it, not taking it sometimes, expressing apathy ‘that I don’t need this anymore, I am having to tolerate so much torture, there is no need for me to live’ something like that, she has a disgust towards herself”

(Counselor, HIV NGO).

A woman who lost her husband soon after diagnosis, and later remarried, described how she came close to committing suicide one night because of abuse related to the intersectional stigma of HIV and widowhood:

“One time my condition was such that I was so disturbed that I was just not able to sleep at night. My husband had died and my aunt was living next door. She used to verbally abuse me so much [in collusion with the in-laws]…use such abusive language, that I was just not able to fall asleep at night. I left the house at three in the morning, locking the door behind me. I had left the house. Then I thought let me do something [suicide]. Then I had this wish, let me do something, what the heck I don’t feel like living anymore. The whole this and that [problems], then I have all this disease in my body, on top of that can I stand listening to so much abuse? I don’t know what came over me…”

(CL014, 36 years).

Loss of income because of HIV stigma and resulting poverty caused women to feel distressed. This was particularly relevant for sex workers, who had no other source of support. Increased poverty led to them having to take up menial jobs and the resulting stigma of poverty (feelings of shame or being demeaned) and the enacted stigma of HIV (verbal abuse) from other sex workers led to some women having thoughts of dying because of experiences of these combined stigmas:

“Maybe some say the body is riddled with worms, some say there is a bad odor…‘You don’t stand next to me’ someone will say, ‘don’t stand next to me and talk’, but they need me only. They need me only, ‘go get two buckets of water for me’ or ‘get me these things from the shop’ or ‘bring me some tea’…They will make you do the work and then [say] all that. Then I feel like if only God took me away right now then I would go right away”

(CL025, 50 years).

### ‘The fear stays with me’

Since non-disclosure was the most important strategy to avoid stigma, including that of domestic violence, women discussed how they constantly lived with a fear of people finding out their status, which they described as *chinta* (worry) and *bhoy* (fear). This affected them mentally as they could never have peace of mind and were always worried. One woman who was separated from her husband described how she could not stop worrying about “someone saying something” if they knew about her HIV status, despite it being almost nine years since her diagnosis:

“Yes, the fear stays, ‘what if someone says something’. Most likely no one will say anything but what if they say something to my face. That fear stays with me. What if they say to my face ‘you don’t come to my house’ and all. That is what. I really don’t think anyone will, but still I live with that fear within me. If they say, ‘don’t come to my house’”

(CL007, 40 years).

Women also had other fears. A woman who had been abandoned by her husband and in-laws because of HIV, and had later had a “stroke”, making it difficult for her to walk, spoke of her fears of the stigma of HIV and disability:

“…I am literate, I can take someone with me and go [to my marital home], but maybe they will say we cannot afford to pay for your keep separately, you have to stay and eat in one place, maybe they can put something in my food, because I cannot walk properly, I cannot do work properly, I am very afraid of that”

(CL001, 46 years).

Several women spoke about their fears for their children. This was mainly the fear of what would happen to their children if they died. One woman described her fear of her sons being driven off by the community if people found out their status. She felt worried and helpless despite being informed that there was law to protect people from HIV related discrimination:

“If people get to know in the neighborhood…the son is growing up, the son who is healthy suppose, maybe he will also not be able to stay. We stay in a rural area, maybe they will not let them stay. Even if laws are enacted, despite there being laws we will not be able to do anything”

(CL020, 32 years).

Such fears of stigmatizing experiences—insults, labeling, or social isolation which women described simply as “worries” and “fear”—were categorized as “trauma” by a clinical psychologist. She thought this “trauma” was greater among women than men:

“The other thing that is there that I have seen as trauma, that is very significant, that is the fear of social isolation. That trauma was found to be pretty intense. It can definitely be categorized as trauma, that social isolation, and they have experienced something. Both males and females, in both cases, but female are more affected”

(Clinical psychologist).

## Stress of intersectional stigma leads to poor physical health

Poor mental health as a result of intersectional stigma, as described above, was perceived by women as having an impact on their physical health. They described three main ways in which this happened: not taking medication, stress related fall in CD4 counts, and the physical effects of stress.

### ‘I won’t take my medicines anymore’

Women often reported throwing away their medication in the period after diagnosis, when stigma and resulting violence against them were at a peak. Not adhering to medication was their way of taking control of a seemingly uncontrollable situation:

“I would not take my medicines, *didi*, I thought I would finish my life… my mother would say ‘you have had your rice, take your medicine’… when mother would go to the bathroom, I would put the pills under the mattress… because I didn’t want to live”

(CL015, 50 years).

Women also reported forgetting to take medications when there was violence at home. One woman described how the stress of HIV-related abuse caused her to neglect her health and medicines even though she actually had every intention to adhere and survive:

“When I would fall asleep after a whole day of fights, I would feel upset and feel down, at that time I was supposed to take it at 9:00…then it was exactly on time…I mean, sometimes he would come back from the truck early in the morning, or at midnight, or at 3:00 or 4:00 in the morning. Maybe he would cause problems and leave or cause problems and stay at home, then there were several times I had gaps in taking medicines”

(CL027, 28 years).

However, many key informants perceived such actions as mental health issues, although they did understand that the issues were reactive to the stress of stigma and violence. HIV-related abuse caused women to drop out of care. A counselor from an HIV NGO seemed to understand women’s language around how such abuse made them feel like they were “not able to go on” and was the reason why so many were lost to follow-up:

“Maybe she is not able to work properly, her health does not permit. I mean if she is always having to bear mental torture or societal torture then that will of course impact other parts of her, the bad effects of it will appear. When health wise she is not able to go on…Maybe at times you will see she is not willing to be linked to care, there are a lot who are becoming LFU (lost to follow-up) cases, not coming for treatment, ‘*didi* I am just not able to take this [torture]’”

(Counselor, HIV NGO).

Another intersection was that of sexual identity and HIV. Transgender women with HIV often faced additional violence due to the combined stigma of their sexual identity and HIV. A public health official felt that such intersectional experiences often caused transwomen to suffer from such poor mental health that they chose to forego treatment:

“The self-esteem is so low that they feel that going on living every day is miserable, so getting treatment means I will go on living, a person who wants to live will come within the treatment regime, someone who does not want to live will not come within the treatment regime”

(Public health official).

### ‘From worrying the CD4 falls’

Chronic stress is known to have a negative effect on the immune system and for people living with HIV this is thought to directly affect CD4 counts. Although measuring CD4 counts was beyond the scope of this work, low CD4 counts due to stress were often an explanation given by some women for why they thought their illness was worse than others.

A woman who had suffered severe violence from her husband and was diagnosed with HIV much before him discussed how the stress of experiencing violence lowered her CD4 counts and was the reason she experienced symptoms much before her husband did. She felt that even now her CD4 fluctuated with her stress levels:

“Now I only have one question…I did not do anything wrong, then how did I get such a big disease. But as soon as my husband had it I understood…his CD4 was good that’s why maybe he got diagnosed later, he didn’t have much mental stress, I had a lot more stress, I could not stand the blow, that’s why I got diagnosed first”

(CL015, 50 years).

Women were also of the opinion that stress due to stigma-related violence caused their CD4 counts to fall, which then caused them to become physically weak. They talked about how a person living with HIV who might be doing well could become sick from the stress associated with negative experiences of multiple stigma and related violence:

“If people think about it [stigma and abuse] they may fall ill, if I am doing well I might fall ill. Because that thing called CD4, then from worrying, if the CD4 is high, then from worrying the CD4 falls, once the CD4 falls, slowly you become weak”

(CL005, 38 years).

Unlike the women interviewees, healthcare providers did not see stress as the key reason for low CD4 counts and attributed it to adherence to ART. An HIV physician, however, attributed the impact on CD4 counts and viral load to non-adherence rather than stress: he observed that fights between couples caused them to “throw the medicines out” and that was a reason why they had low CD4 counts. Although a clinical psychologist acknowledged the role of stress in lowering CD4 counts, much like the women did, she also acknowledged that adherence to ART played a role in improving CD4 counts.

### ‘If I worry my body feels very weak’

Women were asked how their mental health (as a result of all the negative experiences) made them feel physically. Responses included descriptions of several different physical symptoms when they felt worried, stressed, or sad. They described physical symptoms such as loss of appetite and sleep, but also included headaches and other physical symptoms. One woman described severe headaches after HIV-related verbal abuse from her husband:

“If I get stressed, for like half an hour or one hour, my head will hurt so much that I will not be able to tolerate it, but still he will not listen, sometimes he doesn’t listen at all”

(CL006, 26 years).

A woman described how she had lost so much weight due to stress and worry after her husband’s death and subsequent abuse from in-laws that she weighed 30 kilograms (CL030, 39 years). Another widow attributed the presence of blood in her urine to the stress she felt after her husband died: “I mean I had so much stress, my urine was totally red” (CL009, 40 years). A woman described losing her appetite and sleep after episodes of violence from her husband. Overall, she felt that her health was not improving due to the continued stress of violence and having to hide it all because of stigma:

“Yes, then I feel stressed, I am not able to eat properly, I worry, that he is behaving like this with me, I could not sleep, night after night I would also worry. Because I would worry all the time, I mean the disease would always poke at me…I would explain to him but he would not understand”

(CL027, 28 years).

A woman who had been forced to stop taking ART by her abusive husband in the initial years of her diagnosis still complained of health problems, including breathlessness and hallucinations as a result of the long-term nature of HIV-related stress:

“The breathing difficulty worsens, I start to gasp…My head doesn’t ache so much, it just hurts a little, am not able to sleep, stay awake all night and worry. I hallucinate”

(CL008, 45 years).

Another woman attributed her poor physical appearance to the stress of having HIV and all that it entailed:

“No, I mean now due to excessive stress my appearance has become like this. You will see my picture [from before] I am completely different. Before I was totally different, no one could tell that I had this thing in my body”

(CL020, 32 years).

The overall impact of stigma-related violence at home following an HIV diagnosis is illustrated by the following quote, which shows how the different pathways converge to negatively affect mental and physical health for a woman living with HIV:

“I mean if there is tension at home that has an effect. HIV patients are always told to be happy, not worry, so if that space is disrupted, then there is definitely a physical and mental health impact of that…maybe they skip meals, they don’t eat properly, they don’t take their medicines properly, they don’t feel like it”

(Counselor, ART center).

## Discussion

**Fig 1:**
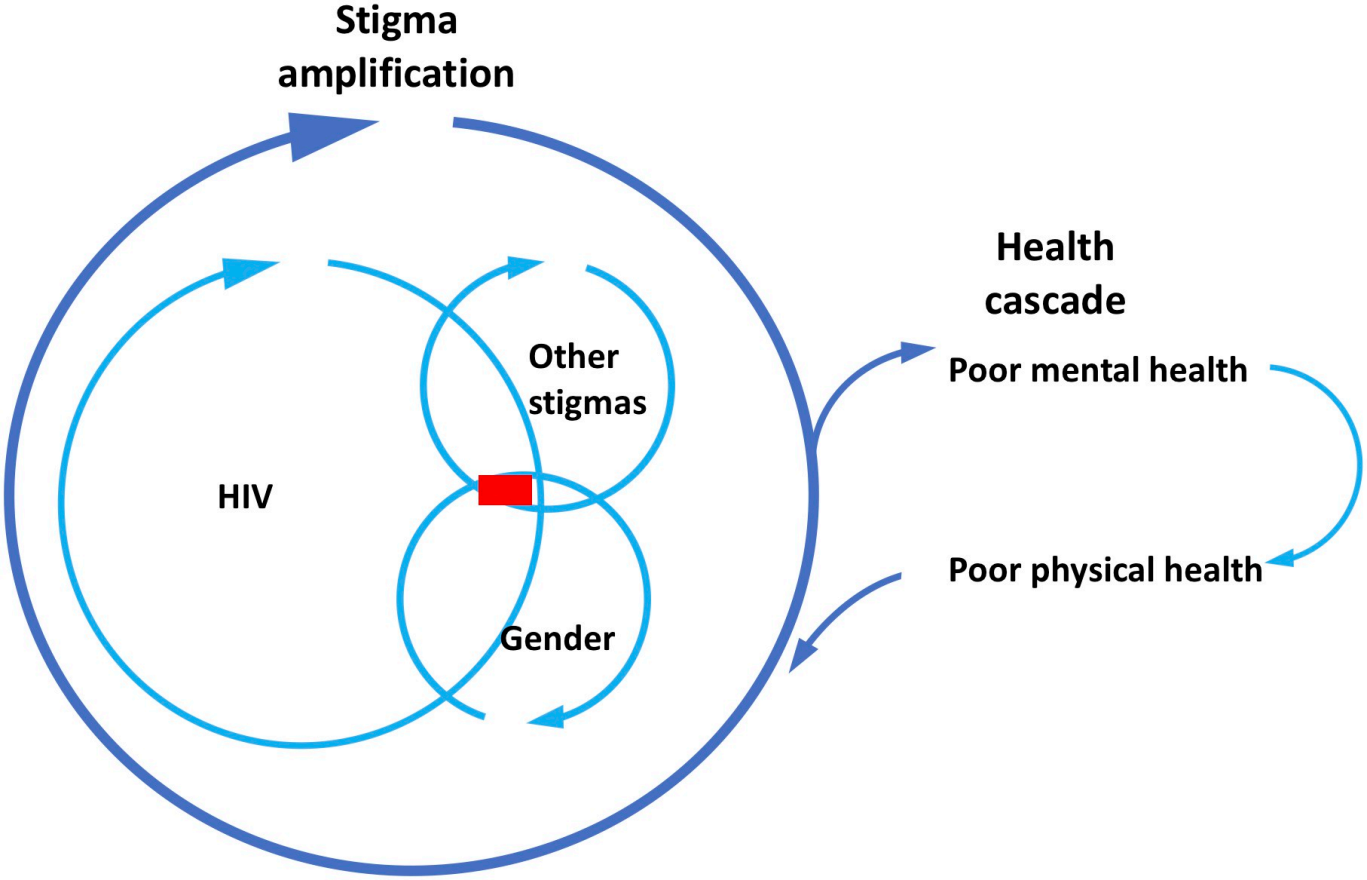
Stigma amplification loop and health cascade for women living with HIV and other marginalized identities.

Women living with HIV often experience worsened stigma and violence associated with their additional marginalized identities [4–6]. In interviews, we found that experiences of intersectional stigma led to poor mental health, which they see as affecting their physical health. We conceptualize this as a stigma amplification loop that feeds into a health cascade (Fig 1): experiences of intersectional stigma and violence lead to poor mental health, which leads in turn to poor physical health, which feeds into the stigma cascade as unwell and unproductive individuals are stigmatized further.

Our findings raise two important issues. First, intersectional stigma affects women’s mental health, which affects their physical health. The effects were perceived as prolonged and stigma has implications for HIV management. Second, labeling women with mental illness attracts some risk and their mental health needs should be addressed through a combination of psychosocial and psychological interventions.

### Intersectional stigma affects women’s mental health, which affects their physical health

Previous studies have shown that intersectional stigma leads to poor mental health [31, 32]. We found that women saw this as affecting their physical health. Low or fluctuating CD4 counts were perceived to be the effects of stigma and violence on their bodies, and were often a reason they attributed for their health to be worse than that of others. Women felt that the mental health impact of intersectional stigma and violence lasted long after they had stopped experiencing them. They continued to feel the fear of anticipated stigma and some even perceived that their current poor physical health was a result of long-term stress on their bodies.

Symptoms such as lack of appetite or sleep and headaches were perceived as mental health effects of stigma and violence. Studies on local understandings of mental health in India have shown that women often conceptualize poor mental health as a consequence of external stressors such as poverty, poor marital relationships, and violence [33, 34]. In a context in which women with clinical depression often present with somatic rather than emotional symptoms [18, 33], expressing physical ailments legitimizes their experiences and affords them a needed break from their regular duties [33]. However, women in our study directly attributed their poor physical health to poor mental health.

Our findings fit with the model proposed by Turan et al., in that we show how intersectional stigma and violence lead to poor mental health: directly through stress processes and indirectly through non-engagement in care and reduced adherence to medication. Despite the widespread availability of free anti-retroviral therapy (ART) and care, women rejected medication because of poor mental health related to intersectional stigma and violence. In the early days of ART, it was argued that widespread rollout would lead to the disappearance of stigma as individuals became healthier [35]. However, stigma continued despite widespread ART availability. Our findings not only add to the body of literature showing that stigma continues to be driven by psychosocial factors—that is, fear of infection and moral blame [36]—but that the mental health effect of HIV and other stigmas makes women reject the ART that is freely available to them. Similar findings have been reported from South Africa, where women reported deliberate non-adherence due to HIV stigma-related violence [37]. An implication is that the mental health impact of intersectional stigma would continue to cause women to be lost to follow-up and ultimately die from a chronic, manageable disease.

### Combined psychosocial and psychological intervention

Our findings highlight the need for integrated stigma reduction and mental healthcare services, which incorporate local understandings and presentations of mental health, for women living with HIV. Women perceived themselves as sad, mentally broken, worried, or troubled, all of which can be mapped onto diagnostic categories of common mental disorders such as anxiety and depression. Neither they nor most of their health providers thought of them as mentally ill. They saw their feelings as reactions to the stigma and violence they had experienced, rather than endogenous. Similar findings have been reported from studies on community mental health in the Sunderban Delta of West Bengal [38]. We see this as a need to treat women’s mental health in a way that addresses their concerns and integrates psychosocial with psychological intervention, as has been recommended for survivors of domestic violence and addressing the social determinants of mental health [39, 40].

Accounts of lived experience are tools for understanding women’s mental health as they allow us to present their collective experiences due to marginalization and loss of status, breakdown of relationships, and violence due to intersectional stigma, rather than presenting them as ill [41]. A diagnosis of mental illness can compound stigma and have adverse effects on Indian women living with HIV by worsening the violence they already experience [6, 24]. Women labelled as mentally ill are at greater risk of divorce in a context in which legislation such as the Hindu Marriage Act, 1955 and Special Marriage Act, 1954, make severe mental illness a ground for nullifying marriage. Husbands and families may exploit this clause to deny women’s right to property, among others [24, 42], and the intersectional stigma of HIV, mental illness, and divorce can open women up to extreme stigmatization such as abandonment by families and communities [43]. Focusing on lived experiences may help women to avoid greater enacted stigma in the form of worsened violence and the internalized stigma that comes with a diagnosis of mental illness.

The lack of labels for women’s feelings has important implications for their support. Not being labeled as mentally ill helped women experience their mental health as an outcome of negative experiences rather than as an additional stigmatized illness to worry about. An understanding of lived experience and the use of non-stigmatizing language should be encouraged in clinical practice. Healthcare providers need to be aware of the risk of labelling, but we recommend that *all* women living with HIV be offered (i) mental health screening and first aid, as well as (ii) psychosocial support in the form of stigma reduction interventions. Women needing additional support should be referred to specialist services so that they can be offered appropriate psychotherapeutic or pharmacological interventions. Very few people in India have their mental health needs met and the mental health component of HIV is often neglected in HIV intervention programs [7]. Considering that women living with HIV may be at increased risk of experiencing poor mental health, it is important that India’s National AIDS Control program does more to increase capacity and deliver appropriate, gender sensitive mental healthcare for women living with HIV. Mental healthcare models that address cumulative trauma [44], by taking into account women’s multiple marginalized identities, may be a promising approach for treatment and care. Healthcare providers must be made aware of women’s alternative expressions of distress in order to provide appropriate support and care without exposing women to additional stigmatization and violence.

### Limitations

Despite its strengths, the study had several limitations. These included a lack of respondents who had dropped out of care. Such women may have had more severe experiences of stigma-related violence and poorer mental health as a result of it, and their experiences were not captured. Since mental illness is stigmatized in this setting, the women interviewed may have minimized their experiences of poor mental health.

## Conclusions

We report two key findings. First, the burden of intersectional stigma leads to a cascade of poor mental and physical health for women living with HIV. Second, it is important to consider the lived experiences of mental health of stigmatized populations such as women living with HIV. All women living with HIV should be screened for mental health and offered mental health first aid. While the label of mental illness should be avoided as far as is reasonable, women needing further support should be offered psychotherapeutic or pharmacologic interventions. However, these must be accompanied by stigma reduction interventions if they are to be successful in addressing the mental health of women living with HIV.

## Supporting information

S1 Checklist Inclusivity in global research. (DOCX)
